# Periprosthetic humeral fractures after shoulder arthroplasty

**DOI:** 10.1530/EOR-2024-0053

**Published:** 2025-06-30

**Authors:** Anna Behrens, Nick Moronga, Milad Farkhondeh Fal, Konrad Mader, Lukas Heilmann, Till Orla Klatte

**Affiliations:** ^1^ Clinic for Shoulder and Elbow Surgery, Vivantes Humboldt Hospital, Berlin, Germany; ^2^ Department for Traumatology and Orthopaedics, University Hospital of Hamburg Eppendorf, Hamburg, Germany; ^3^ Shoulder and Elbow Surgery, Center for General Orthopaedics and Endoprosthetics, Schönklinik Hamburg Eilbek, Hamburg, Germany; ^4^ Orthopaedics Center Hamburg, Hamburg, Germany; ^5^ Division for Hand-, Forearm- and Elbow Surgery, Department for Traumatology and Orthopaedics, University Hospital of Hamburg Eppendorf, Hamburg, Germany; ^6^ Department for Orthopaedic and Trauma Surgery, Albertinen Hospital, Hamburg Schnelsen, Germany

**Keywords:** periprosthetic humerus fracture, revision shoulder arthroplasty, shoulder arthroplasty

## Abstract

- Occurring in 0.5–3% of cases, periprosthetic humerus fractures pose a challenge, necessitating effective management strategies.
- A comprehensive review was conducted using PubMed. Used terms included ‘Periprosthetic humerus fractures; complications; periprosthetic fractures shoulder arthroplasty; periprosthetic humeral fracture treatment; nerve palsy humeral revision arthroplasty; infections after shoulder arthroplasty; postoperative complications AND open reduction AND humeral fractures; allograft AND long humeral stem’. Studies were excluded if they did not meet the actual topic, included more than primary shoulder arthroplasty and/or were in non-English or non-German language.
- Thirty-eight papers with evidence levels ranging from two to three were selected for this review. Various classification systems have been implemented; their validation though was based on studies with only a limited number of patients.
- Risk factors include osteopenia/osteoporosis, rheumatoid arthritis, age, age-related lifestyle and gender.
- Treatment options range from conservative approaches to plate osteosynthesis or revision to a longer stem. Nevertheless, there is a lack of biomechanic studies and randomized-controlled clinical studies; hence, the evidence is low. Complications in revision arthroplasty encompass infections, nonunions, and nerve palsies, highlighting the importance of individualized treatment planning.
- The management of periprosthetic humeral fractures requires careful consideration of risk factors and tailored treatment plans. Existing literature relies on small case series and expert opinions, highlighting the need for further research to establish optimal treatment strategies for these challenging fractures.

## Introduction

The number of performed shoulder arthroplasties has increased during the past 20 years, especially those of reverse shoulder arthroplasty. Therefore, it becomes steadily more important to deal with its possible complications. Especially, the treatment of periprosthetic humerus fractures can be challenging, considering the risk factors of our aging population.

Periprosthetic humeral fractures occur in 0.5–3% of cases with an upward trend (1, 2, 3, 4, 5, 6, 7, 8, 9, 10, 11, 12). Treatment options for periprosthetic humerus fractures include conservative treatment, osteosynthesis with additional plates or revision to a longer stem with or without additional plate osteosynthesis. To date, there is not much data on which treatment modality is favorable for which fracture. Hence, this short review was conducted to present the current data on classifications, risk factors and treatment options with its possible complications on periprosthetic humeral fractures.

## Methods

**Figure 1:**
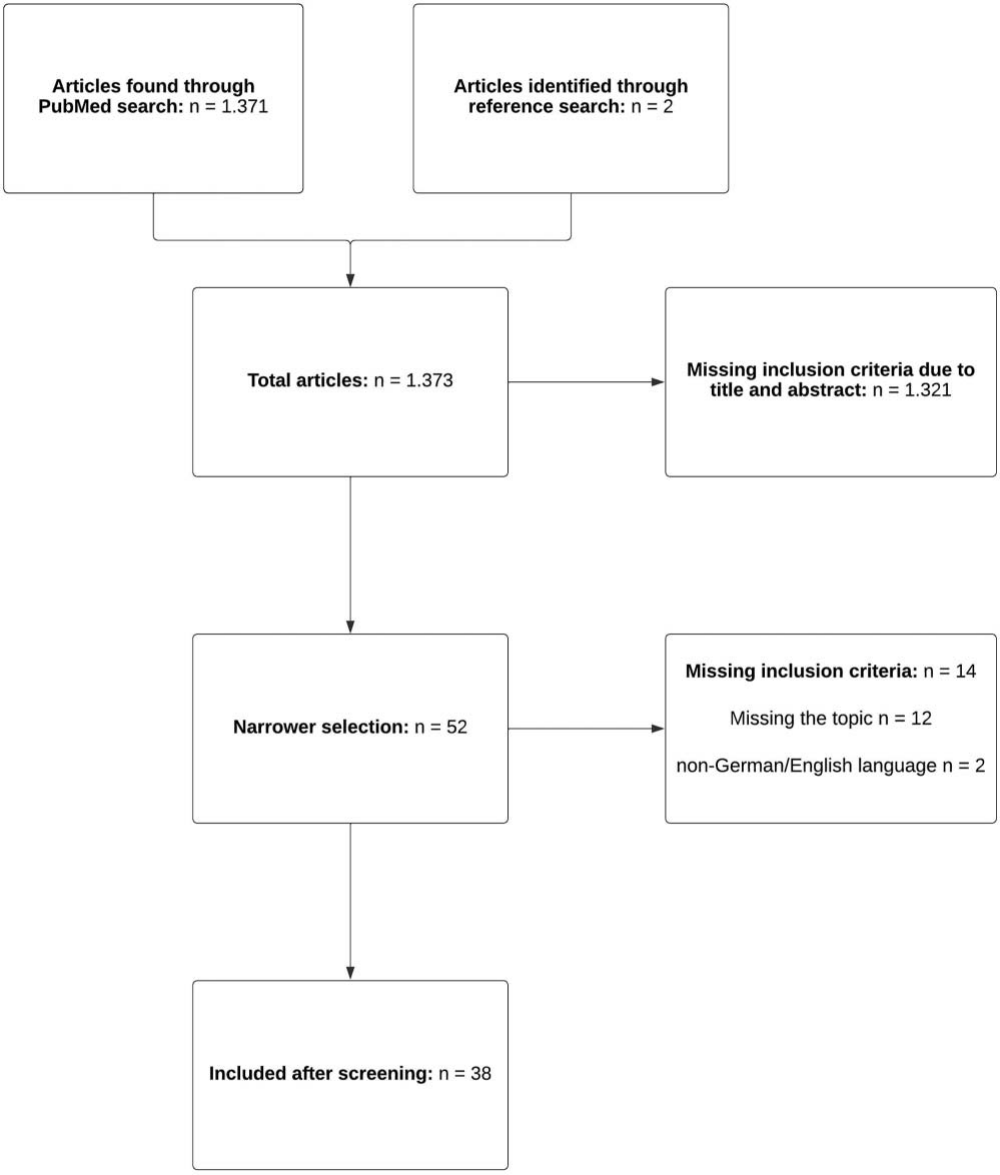
Flowchart: included studies.

This review was created with a keyword search (medical subject headings), manual search and reference search within the medical database PubMed. The used terms were: periprosthetic humerus fractures, complications, periprosthetic fractures shoulder arthroplasty, and periprosthetic humeral fracture treatment. Ninety-four papers (publication years 1996–2023) were identified. A manual search with the term ‘nerve palsy humeral revision arthroplasty’ revealed 28 results and two were used for this review. Furthermore, one manual search with the terms ‘infections after shoulder arthroplasty’ and ‘postoperative complications AND open reduction AND humeral fractures’ revealed 757 and 483 papers. Seven of those were included in our review. The last manual search was performed with the term ‘allograft AND long humeral stem’ and showed nine results, of which two were included in this study. Another two articles were found by reference search. Overall, 52 publications were applicable in a narrower selection (see inclusion and exclusion criteria in Table 1). Of those, 14 publications had to be excluded due to non-English and/or non-German language (*n* = 2) and missing the topic of this review (*n* = 12). Thirty-eight publications were finally applicable for this review (see Fig. 1). These papers varied in their strength of evidence from level two to three. Case reports, research paper and reviews were used for this review. Please note that the used literature has a low number of patients.

**Table 1:** Inclusion and exclusion criteria for literature selection.

Category	Inclusion criteria	Exclusion criteria
Topic	- Articles match the keyword search including: periprosthetic humerus fractures; complications; periprosthetic fractures shoulder arthroplasty; periprosthetic humeral fracture treatment - Articles match manual search including: nerve palsy humeral revision arthroplasty, infections after shoulder arthroplasty, postoperative complications AND open reduction AND humeral fractures, allograft AND long humeral stem	- Articles do not match keyword and manual search - Articles on periprosthetic shoulder fractures other than humerus fractures (e.g., glenoid fractures) - Articles on distal humerus fractures/elbow arthroplasty - Articles on total humerus replacement
Study type	Peer-reviewed articles including meta-analysis, systematic reviews, case-reports, retrospective studies	Non-peer-reviewed articles
Prosthetic type	Anatomic and reverse shoulder prostheses	Non-shoulder-related prostheses
Language	German, English	Non-German/English language

## Results

### Classification systems

In 1995, Wright and Cofield described a conclusive classification with a group of nine periprosthetic humeral fractures based on 499 performed shoulder arthroplasties (2). Three fracture types were described:

- Type A fracture is centered near the tip of the stem and extends proximally.
- Type B fracture is centered near the tip of the stem and extends distally.
- Type C fracture is located distal to the tip of the stem.

Until then, various classification systems for periprosthetic humeral fractures have been introduced. But most of those classifications do not correspond with current circumstances since they are too old to adequately adhere to the newer types of prostheses and their correlated fractures (3). Therefore, previous authors have been emphasizing the need for an updated classification system.

**Figure 2:**
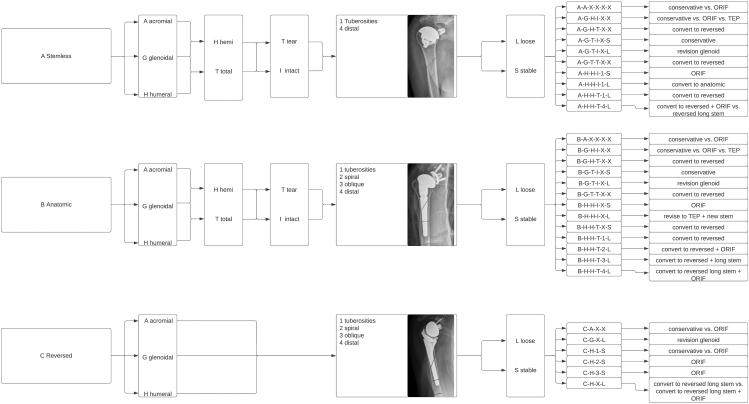
Classification system of Kirchoff *et al.* including therapy algorithm (13, 14).

Kirchhoff *et al.* were the first who published a more complex classification system in 2016 and validated it in 2018 also considering treatment options (13, 14). Kirchhoff *et al.* took into account the prosthetic type, fracture location, glenoid involvement, status of the rotator cuff, and prosthesis stability. Through a comprehensive analysis, they derive therapeutic recommendations, as illustrated in Fig. 2. An advantage of the Kirchhoff classification is the immediate correlation to a designed therapy algorithm in the form of a letter-digit code, which can be beneficial for unexperienced surgeons or complicated fractures (13, 14). In addition to that, the authors achieved a good inter-rater reliability with a Cohen’s kappa of 0.94 (14). A limitation to this algorithm is that the authors validated the classification system and treatment algorithm in only 19 patients, which is likely insufficient to establish generalizability, emphasizing the necessity for larger cohorts. Furthermore, it is essential to note that this validation primarily involves clinical and radiological assessments.

**Figure 3:**
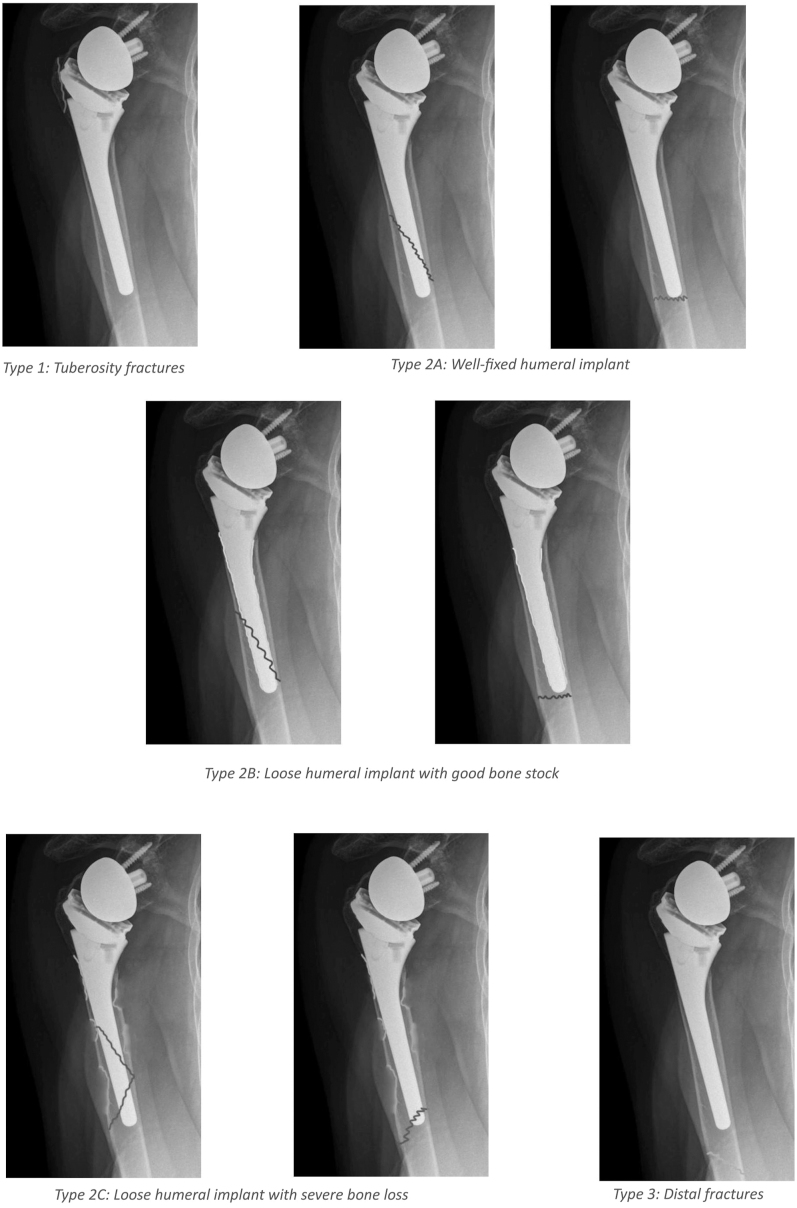
Classification system of Sanchez-Sotelo *et al.* (3).

The latest classification system was published by Sanchez-Sotelo *et al.* 2022 (3). This classification is an extension of the unified classification system and is therefore the internationally recognized classification. The authors describe three fracture types (also see Fig. 3):

- Type 1 are tuberosity fractures.
- Type 2 are peri-implant fractures.
- Type 3 are fractures distal to the prosthesis.

Furthermore, the authors subdivided type 2 fractures in:

- Type 2A: fracture with a well-fixed humeral implant.
- Type 2B: fracture with a loose humeral implant, but adequate bone stock.
- Type 2C: fracture with a loose humeral implant and inadequate bone stock.

In addition to the pure fracture description, all patients undergo a thorough evaluation before therapy (3). This includes their medical history, physical condition, complete imaging, and, if necessary, further examinations (such as infection screening or advanced diagnostics). Based on all collected data, a therapy recommendation is ultimately derived. In summary, conservative treatment is applied in cases of a fixed prosthetic stem with acceptable displacement (type IA, IIA, IIIB, and III). Osteosynthesis is performed for fractures with a fixed prosthetic stem but significant displacement (type IIA and III). In addition to conventional plate osteosynthesis, the option of arthroscopic fixation, for example, in greater tuberosity fractures, is also advocated. Revision with a longer prosthetic stem is necessary in cases of a loosened prosthetic stem (type IB, IIB, and IIC) or in cases of reduced bone stock, where adequate osteosynthesis is not feasible (3). Beyond the purely descriptive classification, it is particularly noteworthy that patient-specific factors are also taken into account. As a result, this treatment algorithm proposed by the authors is likely the most precise. In addition, it is internationally recognized and therefore consistently applicable.

### Risk factors

Risk factors need to be considered before choosing the right treatment option (see Table 2). Osteopenia/osteoporosis is the most important risk factor and is documented very often in patients that suffer from a periprosthetic humeral fracture (1, 2, 4, 5, 12, 15, 16, 17). Rheumatoid arthritis is also a very important risk factor for these fractures (1, 2, 4, 9, 12, 17). Furthermore, studies show a correlation of a higher prevalence of rheumatoid arthritis and osteoporotic bone in women (1, 4, 11). Other risk factors are the increasing age (average 63–77 years) and osteoarthrosis (7, 8, 15, 18). In addition, the claim for a higher health-related quality of life including a more active life in general with a higher risk of trauma and the female gender are the risk factors for periprosthetic humeral fractures in both, anatomical and reverse total shoulder arthroplasty (TSA) (2, 4, 5, 7, 8, 10, 12, 18). In addition to that, Athwal *et al.* reported that women were three times more likely to sustain fractures already during shoulder arthroplasties (12). Intraoperatively, it is therefore important to handle the humerus with care because rough reaming or extensive external rotation can lead to fractures (1, 12, 17). Finally, Athwal *et al.* described revision shoulder arthroplasty as a risk factor itself (12).

**Table 2:** Risk factors for periprosthetic humerus fractures.

Study	Study type	Patients, *n*		Risk factor
		Postop	Intraop	
Boyd *et al.* (4)	RS	7		Osteoporosis, rheumatoid arthritis, active life, female gender
Wright & Cofield (2)	RS	9		Osteoporosis, rheumatoid arthritis, active life, female gender
Campbell *et al.* (1)	RS	5	16	Osteoporosis, rheumatoid arthritis, rough reaming or extensive external rotation
Worland *et al.* (5)	RS	6		Osteoporosis, active life, female gender
Kumar *et al.* (15)	RS	16		Osteoporosis, age
Athwal *et al.* (12)	RS		45	Osteoporosis, rheumatoid arthritis, active life, female gender, rough reaming or extensive external rotation, revision shoulder arthroplasty, press-fit humeral implants
Sommacal *et al.* (16)	CR	1		Osteoporosis
Wolf *et al.* (7)	RS	8		Age, active life, female gender
Singh *et al.* (11)	MRR	25	47	Female sex, underlying diagnosis (most often posttraumatic arthritis), higher Deyo–Charlson Index, ASA class of 3 or 4
Sewell *et al.* (18)	RS	22		Age, active life, female gender
Garcia-Fernandez *et al.* (8)	RS	4	3	Age, active life, female gender
Wagner *et al.* (10)			36	Active life, female gender, removal of components, poor remaining bone stock, instability in history, prior hemiarthroplasty

ORIF, open reduction and internal fixation; OSS, Oxford Shoulder Score; RS, retrospective study; CR, case report; MRR, medical record report.

### Treatment options

To treat a periprosthetic humeral fracture three different treatment options are described: conservative treatment, osteosynthesis with a plate and/or revision to a longer stem. Table 3 provides an overview of all included studies showing treatment options und outcome of periprosthetic humerus fractures.

**Table 3:** Included clinical studies on the treatment and outcome of periprosthetic humerus fractures.

Study	Year	Study type	Patients, *n*	Treatment options: *n*	Outcome parameters		Complications: *n*
					Mean time to union	Other	
Boyd *et al.* (4)	1992	RS	7	Surgery: 5 (ORIF with plate: 2; ORIF with long stem: 3); conservative treatment: 2	5 months		12 (decreased ROM: 6; residual pain: 6)
Wright & Cofield (2)	1995	RS	9	Surgery: 5 (long stem: 2; ORIF: 3); conservative treatment: 4	4–6 months*	Fracture union: *n* = 8	6 (radial nerve palsy: 2; poor ROM: 2; persistent pain: 2)
Campbell *et al.* (1)	1998	RS	21	Revision arthroplasty: 16 (used stem length at least 3 cortical diameters distal to fracture site); conservative treatment: 5	0.8 months		10 (hardware associated: 2; soft tissue associated: 6; delayed rehabilitation: 1; neuropraxia: 4; nonunion: 1)
Worland *et al.* (5)	1999	RS	6	Surgery: 5 (long-stem + allograft: 4; ORIF + allograft: 1); conservative treatment: 1	3.3 months	UCLA score: 25.8	n/a
Kumar *et al.* (15)	2004	RS	16	Surgery: 10 (ORIF: 5; long stem: 5); conservative treatment: 6	278 days	ROM: ABD = 107°, ER = 43°	Postoperative infection: 1; revision surgery: 1
Groh *et al.* (22)	2008	RS	15	Surgery: 10 (long-stem + cerclage: 6; ORIF + cerclage: 2; cerclage wires: 2); conservative treatment: 5	11 weeks	Forward elevation: 124°	none
Athwal *et al.* (12)	2009	RS	45	ORIF: 23; revision arthroplasty: 13; conservative: 9		Fracture healing (mean): 4.25 months; forward elevation = 108°; ER = 39°	14 (plexus/nerve palsy: 6; displaced/re-fracture: 5; soft tissue associated: 2; glenoid arthritis: 1)
Sommacal *et al.* (16)	2009	CR	1	Long-stem: 1	6 weeks		n/a
Wolf *et al.* (7)	2012	RS	8	Surgery: 6 (ORIF: 4; revision arthroplasty: 2); conservative treatment: 2		Fracture union after six months: *n* = 5	6 (radial nerve palsy: 2; non-union: 3; osteosynthesis failure: 1)
Sewell *et al.* (18)	2012	RS	22	Surgery: 20 (long-stem: 12; standard stem: 8); conservative treatment: 2	27 weeks	OSS (mean) = 25	10 (nerve palsy: 2; soft tissue associated: 3; hardware associated: 5)
Andersen *et al.* (23)	2013	RS	36	Surgery: 36 (ORIF: 17 (allograft: 8); long-stems: 14; short-stems: 5)	7.2 months	ASES: 50.3; *n* = 35/36 fractures healed	14 (ORIF: 7; revision arthroplasty: 7)
Owens *et al.* (28)	2013	RS	12	Surgery (long stem): 12	n/a		1 (non-union)
Garcia-Fernandez *et al.* (8)	2015	RS	7	Surgery: 6 (ORIF: 5 (allograft: 1); long-stem + cerclage: 1); conservative treatment: 1	2–6 months		2 (radial nerve palsy)
Wagner *et al.* (10)	2015	RS	36	Surgery: 36 (press-fit stem: 20; cemented stem: 8; internal fixation: 8)		ASES (points): 65.0; SST: 6.2; ABD: 115°	11 (postop humerus fracture: 5; humeral lucency: 3; glenoid loosening: 2; instability: 1)
Jaeger *et al.* (6)	2017	CS	17	Surgery: 17 (revision arthroplasty: 4 (used stem length: at least 6 cm distal the humeral fragment); plate osteosynthesis: 13)	n/a		1 nonunion
Geβmann *et al.* (21)	2019	CS	40	Surgery: 40 (plate osteosynthesis: 30; long-stem and RTSA: 10)	n/a		8 (infections: 3; radial nerve palsies: 3; pseudarthrosis: 2)

* Median value.

RS, retrospective study; CR, case report; CS, case series; ORIF, open reduction and internal fixation; OSS, Oxford Shoulder Score; ASES, American Shoulder and Elbow Surgeons – Score; SST, Simple Shoulder Test – Score; SSV, Subjective Shoulder Value; CSS, Constant Shoulder Score; RTSA, reverse shoulder total shoulder arthroplasty; ROM, Range of motion; mo, months; n/a., not applicable; ABD, Abduction; ER, external rotation.

Kirchhoff *et al.* published a therapy algorithm for the different types of fractures. Those can be found in Fig. 2 (13, 14). Nevertheless, as mentioned before, this algorithm was validated on only 19 patients.

According to the current literature, conservative treatment is indicated in type B and C fractures regarding the classification of Wright and Cofield, respectively, type 1A, 2A and 3 fractures according to Sanchez-Sotelo *et al.* if the residual displacement is acceptable (1, 2, 3, 5, 15, 18, 19, 20). It is performed by immobilizing the arm in a brace. One of the biggest complications is a nonunion of the fracture, which can require further treatment options (4, 15). On the other side, it is a good option for patients with underlying health conditions increasing their intraoperative risk factors (19). Kumar *et al.* stated that an attempt of 3 months for conservative treatment is acceptable if the implant is well-fixed (15). In their study, the authors propose a conversion to surgery after an unsuccessful conservative therapy period of 3 months and a remaining nonunion (3, 15). Wright and Cofield described nine periprosthetic humeral fractures after TSA, with eight being treated conservatively (2). Although, only four out of these eight fractures healed properly (2). Campbell *et al.* reported 21 periprosthetic fractures, of which five were treated nonoperatively; four out of these five fractures healed successfully within a mean of 3.5 months (1).

The treatment with plate osteosynthesis (with or without additional k-wire-cerclages) is currently indicated in type B and C fractures according to Wright and Cofield or type 2A and also type 3 fractures referring to Sanchez-Sotelo *et al.* as long as the humeral implant is well-fixed and the displacement indicates surgery (2, 3, 20). The current literature does not provide sufficient information on the amount of displacement that can be tolerated, making it impossible to give a clear recommendation. The compression plate, which was frequently used in the past, now plays only a minor role due to implant failure. Periprosthetic fractures should be addressed with angular stable plate systems (3). There is no consensus on which angular stable plate should be used: Kirchhoff *et al.* proposed the use of polyaxial plates over the generally used locking plates (19). Garcia-Fernandez *et al.* preferred osteosynthesis using locking compression plates (LCP) (8). According to Sanchez-Sotelo *et al.* type 1 fractures may benefit from open reduction and internal fixation (ORIF) using periarticular proximal humerus fracture plates (3). Monoaxial plates (for example, LCP) enable the combination of compression in the oval sliding portion with angular stable locking in the threaded portion of the plate hole, whereas polyaxial plates (NCB, non-contact bridging) allow polyaxial screw placement, where fixation to the plate occurs secondarily. This gives the surgeon the ability to accurately assess the patient’s bone quality. Although locking plate generally allows putting the drill sleeves percutaneously to minimize soft tissue trauma, these fractures need good visualization in order to protect surrounding tissue, especially the radial nerve (3, 7, 21). When placing the plate, the fracture should be bridged by a minimum of 2 cortical diameter of the humerus (14). A key factor is that the screws are aligned precisely and to use bicortical screws, if possible, alternatively monocortical locking screws can be used in the proximal part of the humerus (3).

The last treatment option is a revision to a longer stem. It is indicated if the prosthesis is loose and/or if the bone stock is very poor (fracture type 1B, 2B and 2C regarding to Sanchez-Sotelo *et al.* (3)) (1, 2, 3, 4, 5, 7, 18). It is important to choose the right size of the new and most often longer stem since it is important for fracture healing that the healthy part of the humerus is secure and hoop-stressing is limited (1). There is not much data on the recommended length for the new stem (1, 6, 16, 18, 20). Case studies and expert opinions suggest an average length of 2–3 cortical diameters (around 6 cm), surpassing the fracture site to achieve enough fracture stabilization and to lower the risk of refracturing (1, 6, 18, 22). Andersen *et al.* reported 36 periprosthetic humeral fractures, 14 fractures were treated by a long stem prosthesis and all but one healed after a mean time of 8 months, proposing the standard use of a long revision stem (23). Nevertheless, if the patient has adequate bone stock, a standard stem might also be an option (3). In the end, the fracture itself impacts the decision making whether to use a standard or long revision stem.

The additional use of cement depends on the condition of the humerus, especially the medullary cavity and bone stock in general. If the surgeon decides to use cement, it is very important that no cement fluid is leaking out of the fracture gap in order to prevent postoperative complications such as tissue damage, especially nerve damage, and nonunion (6).

In case of poor bone stock and/or structural bone deficits, the treatment of the periprosthetic humeral fracture can generally become challenging despite the right treatment option. Additional strut allografts are a good option to stabilize the fracture site. Only few data exist on the use of allografts in revision shoulder arthroplasty. Gohlke *et al.* published a treatment strategy of bone defects type 3 and 4 of their own classification to be fixed with a long revision stem and allograft as an intramedullary load carrier (24). Their study showed that the use of allografts is biomechanically favorable in bone defects greater than 5 cm (24). Trompeter *et al.* presented a short case series in which all patients were treated by long stem and strut allografts. The patients achieved good clinical outcomes comparable to other publications without the use of allografts (25). Berkes *et al.* presented 11 cases of complex proximal humeral fractures. All patients were treated with fibular allograft, long lateral locking plate and additional screws and every patient had a good clinical outcome, fracture union, was pain free and had no restriction in the range of motion (26). In a meta-analysis, Nie *et al.* analyzed the effect of fibular strut augmentation for ORIF in humeral fractures in general. They compared eight studies with 596 patients overall (27). They concluded that a significant association between fibular strut allografts and good clinical outcomes, e.g. functional recovery and low risk of complications, exists (27). Allografts have the advantage of supporting the fracture site by increasing the possible fracture load (26). Due to the biological character of the allograft, they can also incorporate with the humeral shaft (25). On the other hand, it must be noted that allografts have a low risk of disease transfer to the recipient and a general infection risk (24, 25). In addition, the access to these allografts is limited in some countries due to the national guidelines of the government (24). In the end, revision surgery using additional strut allografts should be left to experts.

### Complications of revision surgery

In a series including 612 revision shoulder arthroplasties with 110 (18%) patients treated with an intermediate or long stem, Owens *et al.* reported 13 (16.3%) intraoperative complications including cement extrusion, distal cortical perforation of the stem, nonunions and deep infections (28). Wagner *et al.* presented 36 periprosthetic humeral fractures out of 230 revision reverse shoulder arthroplasties (10). Most were stabilized by a press-fit implant (20) and cemented stem (8). They reported postoperative fractures (*n* = 2), glenoid loosening (*n* = 2) and loss of stability (*n* = 1) (10). Campbell *et al.* stated a complication rate of 43% including temporary neuropraxia of the radial nerve, loss of fracture fixation, delayed and nonunion (partly with hardware failure) (1).

Nerve palsy is a rare complication but can still occur while implanting humeral stems in general. Out of 417 performed total shoulder arthroplasties, Lynch *et al.* reported 18 patients (4.3%) with nerve palsy after the primary stem implantation (29). In revision arthroplasty, it can be more frequent due to possible adhesions during exposure of the fracture site, especially at the humerus shaft, and a more frequent use of a circular stabilization, e.g. cerclage wires (30, 31). The current literature also shows that nerve palsy is most common due to increased traction forces during surgery and cement extrusion, if cement is used.

Furthermore, infections should be considered as a major complication in revision surgery. In several trials, authors have stated that the overall infection rate in ORIF and shoulder arthroplasties varies between 1.2 and 1.6% (32, 33, 34, 35). In general, ORIF seems to be the more protective option to avoid infections compared to anatomic or reverse arthroplasty (36). Using standardized algorithms can help in reducing the infection rate. It is important to monitor the patient for any clinical signs of infections, e.g., pain, stiffness, fever, night sweats and chills. In addition, strict blood count monitoring is advisable by focusing on leukocytes and c-reactive protein. But note that these parameters may also be elevated for other reasons and should therefore not be the sole basis for a therapy decision. It is important to cultivate samples, for example, preoperatively by joint aspiration or arthroscopic sampling (37, 38). However, it should be noted that low-virulence bacteria are often present in the shoulder joint and these may not be recognizable in the aspirate compared to infections in other joints (37). Hence, a two-step revision with culture sampling with an odd number of samples from various wound layers should be considered.

## Conclusion

The treatment of periprosthetic humeral fractures is challenging and should be well-planned. To date, only few studies exist on how periprosthetic humeral fractures should be treated. Treatment recommendations rely on small case series and expert opinions. Risk factors such as osteopenia/osteoporosis, rheumatoid arthritis, age and gender need to be considered for choosing the best treatment option. In periprosthetic humeral fractures with a well-fixed stem therapy options such as ORIF with plate osteosynthesis or nonoperative management may be viable options, depending on the fracture’s characteristics and patient-specific considerations. Conversely, a loose stem or poor bone quality often requires stem revision. When selecting a surgical route, it is crucial to acknowledge potential complications, including nerve palsy, intraoperative fractures, nonunions, and cement extrusion. A possible fracture site support by strut allograft can be taken into account. Especially, revision to a long-stemmed prosthesis is not investigated well enough. The current literature recommends that the revision stem should span the fracture site by at least 2 to 3 cortical diameters (approximately 6 cm). This recommendation is primarily rooted in expert opinions and limited case studies. To our knowledge, there are no biomechanical studies investigating this problem. In conclusion, addressing the complex issue of periprosthetic humeral fractures requires a joint effort to bridge the current knowledge gap through comprehensive biomechanical research and randomized trials.

## ICMJE Statement of Interest

The authors declare that there is no conflict of interest regarding the publication of this work. There are no financial, personal, or professional affiliations that could be perceived as influencing the objectivity of the work reported herein.

## Funding Statement

The authors declare that there was no external funding or financial support for the research, authorship, and/or publication of this work.

## References

[1] Campbell JT, Moore RS, Iannotti JP, et al. Periprosthetic humeral fractures: mechanisms of fracture and treatment options. J Shoulder Elbow Surg 1998 7 406–413. (10.1016/s1058-2746(98)90033-7) 9752653

[2] Wright TW & Cofield RH. Humeral fractures after shoulder arthroplasty. J Bone Joint Surg Am 1995 77 1340–1346. (10.2106/00004623-199509000-00008) 7673283

[3] Sanchez-Sotelo J & Athwal GS. Periprosthetic postoperative humeral fractures after shoulder arthroplasty. J Am Acad Orthop Surg 2022 30 e1227–e1239. (10.5435/jaaos-d-21-01001) 36026696

[4] Boyd AD, Thornhill TS & Barnes CL. Fractures adjacent to humeral prostheses. J Bone Joint Surg Am 1992 74 1498–1504. (10.2106/00004623-199274100-00008) 1469009

[5] Worland RL, Kim DY & Arredondo J. Periprosthetic humeral fractures: management and classification. J Shoulder Elbow Surg 1999 8 590–594. (10.1016/s1058-2746(99)90095-2) 10633894

[6] Jaeger M, Maier D, Izadpanah K, et al. Prothesenwechsel bei periprothetischer Humerusfraktur. Operat Orthop Traumatol 2017 29 492–508. (10.1007/s00064-017-0521-9) 29063283

[7] Wolf H, Pajenda G & Sarahrudi K. Analysis of factors predicting success and failure of treatment after type B periprosthetic humeral fractures: a case series study. Eur J Trauma Emerg Surg 2011 38 177–183. (10.1007/s00068-011-0145-y) 26815835

[8] García-Fernández C, Lópiz-Morales Y, Rodríguez A, et al. Periprosthetic humeral fractures associated with reverse total shoulder arthroplasty: incidence and management. Int Orthop 2015 39 1965–1969. (10.1007/s00264-015-2972-7) 26318881

[9] Della Rocca GJ, Leung KS & Pape HC. Periprosthetic fractures: epidemiology and future projections. J Orthop Trauma 2011 25 S66–S70. (10.1097/bot.0b013e31821b8c28) 21566478

[10] Wagner ER, Houdek MT, Elhassan BT, et al. What are risk factors for intraoperative humerus fractures during revision reverse shoulder arthroplasty and do they influence outcomes? Clin Orthop Relat Res 2015 473 3228–3234. (10.1007/s11999-015-4448-x) 26162412 PMC4562920

[11] Singh JA, Sperling J, Schleck C, et al. Periprosthetic fractures associated with primary total shoulder arthroplasty and primary humeral head replacement. J Bone Joint Surg Am 2012 94 1777–1785. (10.2106/jbjs.j.01945) 23032588 PMC3448303

[12] Athwal GS, Sperling JW, Rispoli DM, et al. Periprosthetic humeral fractures during shoulder arthroplasty. J Bone Joint Surg Am 2009 91 594–603. (10.2106/jbjs.h.00439) 19255219

[13] Kirchhoff C, Kirchhoff S & Biberthaler P. Klassifikation periprothetischer schulterfrakturen. Unfallchirurg 2016 119 264–272. (10.1007/s00113-016-0159-3) 26992712

[14] Kirchhoff C, Beirer M, Brunner U, et al. Validation of a new classification for periprosthetic shoulder fractures. Int Orthop 2018 42 1371–1377. (10.1007/s00264-018-3774-5) 29353316

[15] Kumar S, Sperling JW, Haidukewych GH, et al. Periprosthetic humeral fractures after shoulder arthroplasty. J Bone Joint Surg Am 2004 86 680–689. (10.2106/00004623-200404000-00003) 15069130

[16] Sommacal R, Bloch HR, Ghidelli A, et al. Comminuted periprosthetic humeral fracture after reverse shoulder prosthesis. Musculoskelet Surg 2009 93 83–87. (10.1007/s12306-009-0013-7) 19711175

[17] Cuomo F & Checroun AJ. Avoiding pitfalls and complications in total shoulder arthroplasty. Orthop Clin North Am 1998 29 507–518. (10.1016/s0030-5898(05)70025-0) 9706296

[18] Sewell MD, Kang SS, Nawfal A-H, et al. Management of peri-prosthetic fracture of the humerus with severe bone loss and loosening of the humeral component after total shoulder replacement. J Bone Joint Surg Br 2012 94-B 1382–1389. (10.1302/0301-620X.94B10.29248) 23015565

[19] Kirchhoff C, Brunner U & Biberthaler P. Periprosthetic humeral fractures. Unfallchirurg 2016 119 273–280. (10.1007/s00113-016-0161-9) 27008215

[20] Gebrelul A, Green A, Schacherer T, et al. Periprosthetic humerus fractures: classification, management, and review of the literature. Ann Joint 2018 3 49. (10.21037/aoj.2018.06.02)

[21] Geßmann J, Königshausen M, Schildhauer TA, et al. Periprothetische Humerusfraktur – von der plattenosteosynthese bis zum Prothesenwechsel. Operat Orthop Traumatol 2019 31 84–97. (10.1007/s00064-019-0591-y) 30820585

[22] Groh GI, Heckman M, Wirth MA, et al. Treatment of fractures adjacent to humeral prostheses. J Shoulder Elbow Surg 2008 17 85–89. (10.1016/j.jse.2007.05.007) 18069012

[23] Andersen JR, Williams CD, Cain R, et al. Surgically treated humeral shaft fractures following shoulder arthroplasty. J Bone Joint Surg Am 2013 95 9–18. (10.2106/jbjs.k.00863) 23283369

[24] Gohlke F, Berner A & Abdelkawi A. Humerale Knochendefekte in der Revisionsendoprothetik. Die Orthopädie 2023 52 98–108. (10.1007/s00132-022-04335-5) 36651969

[25] Trompeter A & Gupta R. The management of complex periprosthetic humeral fractures: a case series of strut allograft augmentation, and a review of the literature. Strategies Trauma Limb Reconstr 2013 8 43–51. (10.1007/s11751-013-0155-x) 23457000 PMC3623919

[26] Berkes MB, Little MTM, Lazaro LE, et al. Intramedullary allograft fibula as a reduction and fixation tool for treatment of complex proximal humerus fractures with diaphyseal extension. J Orthop Trauma 2014 28 e56–e64. (10.1097/bot.0b013e31829a346d) 24561540

[27] Nie W, Wang Z, Gu F, et al. Effects of fibular strut augmentation for the open reduction and internal fixation of proximal humeral fractures: a systematic review and meta-analysis. J Orthop Surg Res 2022 17 322. (10.1186/s13018-022-03211-4) 35729668 PMC9210738

[28] Owens CJ, Sperling JW & Cofield RH. Utility and complications of long-stem humeral components in revision shoulder arthroplasty. J Shoulder Elbow Surg 2013 22 e7–e12. (10.1016/j.jse.2012.10.034) 23352057

[29] Lynch NM, Cofield RH, Silbert PL, et al. Neurologic complications after total shoulder arthroplasty. J Shoulder Elbow Surg 1996 5 53–61. (10.1016/s1058-2746(96)80031-0) 8919443

[30] Akhtar A & Ng CY. Cement extrusion and radial nerve palsy during revision shoulder and elbow arthroplasty: beware of the cortical breach. J Clin Orthopaedics Trauma 2021 16 226–229. (10.1016/j.jcot.2021.02.013) PMC7920133 33717959

[31] Fu MC, Hendel MD, Cheng X, et al. Surgical anatomy of the radial nerve in the deltopectoral approach for revision shoulder arthroplasty and periprosthetic fracture fixation: a cadaveric study. J Shoulder Elbow Surg 2017 26 2173–2176. (10.1016/j.jse.2017.07.021) 28939334

[32] Boileau P, Sinnerton RJ, Chuinard C, et al. Arthroplasty of the shoulder. J Bone Joint Surg Br 2006 88-B 562–575. (10.1302/0301-620x.88b5.16466) 16645099

[33] Strickland JP, Sperling JW & Cofield RH. The results of two-stage re-implantation for infected shoulder replacement. J Bone Joint Surg Br 2008 90-B 460–465. (10.1302/0301-620x.90b4.20002) 18378920

[34] Saltzman MD, Marecek GS, Edwards SL, et al. Infection after shoulder surgery. J Am Acad Orthop Surg 2011 19 208–218. (10.5435/00124635-201104000-00005) 21464214

[35] Robinson CM, Stirling PHC, Goudie EB, et al. Complications and long-term outcomes of open reduction and plate fixation of proximal humeral fractures. J Bone Joint Surg Am 2019 101 2129–2139. (10.2106/jbjs.19.00595) 31800426

[36] Cvetanovich GL, Chalmers PN, Verma NN, et al. Open reduction internal fixation has fewer short-term complications than shoulder arthroplasty for proximal humeral fractures. J Shoulder Elbow Surg 2016 25 624–631.e3. (10.1016/j.jse.2015.09.011) 26686759

[37] Lemmens L, Geelen H, Depypere M, et al. Management of periprosthetic infection after reverse shoulder arthroplasty. J Shoulder Elbow Surg 2021 30 2514–2522. (10.1016/j.jse.2021.04.014) 33895302

[38] Sperling JW, Kozak TKW, Hanssen AD, et al. Infection after shoulder arthroplasty. Clin Orthop Relat Res 2001 382 206–216. (10.1097/00003086-200101000-00028) 11153989

